# The effects of meditation on length of telomeres in healthy individuals: a systematic review

**DOI:** 10.1186/s13643-021-01699-1

**Published:** 2021-05-21

**Authors:** Nirodhi N. Dasanayaka, Nirmala D. Sirisena, Nilakshi Samaranayake

**Affiliations:** 1 Research Promotion and Facilitation Centre, Faculty of Medicine, University of Colombo, Colombo, 00800 Sri Lanka; 2 Human Genetics Unit, Department of Anatomy, Faculty of Medicine, University of Colombo, Colombo, 00800 Sri Lanka; 3 Department of Parasitology, Faculty of Medicine, University of Colombo, Colombo, 00800 Sri Lanka

**Keywords:** Meditation, Telomere length, Healthy participants, Case-control studies, Randomized controlled trials, Systematic review

## Abstract

**Background:**

Meditation-based practices have been suggested to result in many biological benefits which include reduction of attrition of telomeres, the protective nucleotide-protein complexes at termini of eukaryotic chromosomes. This systematic review evaluated the effects of meditation on telomere length (TL) in healthy adults.

**Methods:**

Randomized controlled trials (RCTs) and observational studies conducted to determine the effects of meditation on TL in healthy individuals, published up to July 2020 were retrieved by searching seven electronic databases (PubMed, Scopus, PsycINFO, EMBASE, Cochrane Library, CINAHL and Google Scholar). The methodological quality of RCTs and observational studies was assessed using the Cochrane Collaboration Risk of Bias Tool and Joanna Briggs Institute critical appraisal checklist, respectively. The data was synthesized narratively and the effect estimates of TL in the RCTs were synthesized using alternative methods as a meta-analysis was not conducted. The certainty of evidence was classified according to the GRADE system.

**Results:**

A total of 1740 articles were screened. Five studies comprising two RCTs and three case-control studies (CCS) were included in the final review based on the inclusion and exclusion criteria. The combined sample consisted of 615 participants with 41.7% males. Average age of participants was 47.7 years. One CCS and one RCT reported significant beneficial effects of meditation on TL while the two remaining CCS and the RCT showed positive effects of meditation on TL which were not significant. For all CCS and one RCT, the methodological quality was high while the remaining RCT was of moderate quality. The quality of evidence for the primary outcome was moderate in RCTs.

**Conclusion:**

The effect of meditation on TL per se is still unclear. Strictly designed and well-reported RCTs with larger sample sizes are required to provide evidence of higher quality.

**Systematic review registration:**

The protocol of this review was registered with the International Prospective Register of Systematic Reviews (PROSPERO) database (registration number: CRD42020153977).

**Supplementary Information:**

The online version contains supplementary material available at 10.1186/s13643-021-01699-1.

## Background

Meditation is an ancient technique which promotes a sense of calm and heightened awareness and improves general physical well-being [1]. Techniques incorporating these teachings have now become widely popular for building resilience to stressors of everyday life and illness [2]. Currently, the term meditation broadly refers to a number of varying techniques such as Breathing, Body-Scan, Walking, Zen, Vipassana, Loving-kindness (LKM), Mindfulness, and Concentrative meditation. A growing body of evidence has highlighted biological effects that may be brought upon by meditation that is beneficial in health and disease.

One such area of expanding interest is cellular ageing affected by the length of telomeres. Telomeres are protective DNA end caps of chromosomes, dysfunction or loss of which can lead to genomic instability, cellular apoptosis and senescence. Length of telomeres is regulated by cellular telomerase enzyme levels and multiple other biological factors. In addition to being considered a biomarker of the natural ageing process [3], short TL has been demonstrated to be a risk or prognostic marker in several disease conditions which are primarily associated with an age related onset [4,6].

Meditation may bring about biological benefits by reducing psychological distress [7] and influencing gene expression and pathways central to regulating cellular oxidative stress [8]. A meta-analysis conducted on studies which compared a mindfulness-based practice with a control condition in variable populations concluded that meditation-based interventions may impact TL and that a longer duration of meditation practice may favour maintenance of telomere length [9]. Another similar meta-analysis has shown that mindfulness meditation leads to increased telomerase activity [10].

However, the evidence of these analyses is influenced by the heterogeneous study populations and combined interventions, where changes in TL may be influenced by other stress-relieving activities or the presence of disease conditions which themselves may affect the process of telomere shortening. Further, novel data from interventional studies, describing the effects of meditation on TL, have been recently published [11, 12]. A clear understanding of changes in TL conferred by meditation-based practices in otherwise healthy persons would not only add to the knowledge on cellular ageing but also strengthen the scientific basis of use of these complimentary approaches in the management of many chronic and inflammatory disease conditions. This study reviewed the evidence from RCTs and observational studies on TL in healthy adults who have followed meditation-based practices.

## Methods

The protocol of this review was registered with the International Prospective Register of Systematic Reviews (PROSPERO; ID No: CRD42020153977) and is reported in accordance with the Preferred Reporting Items for Systematic Reviews and Meta-Analyses Protocol (PRISMA) statement [13].

A systematic literature search was carried out for articles published in English in PubMed, Scopus, PsycINFO, EMBASE, Cochrane Library, CINAHL and Google Scholar databases from their inception to 30 July 2020 (see Additional file 1). Since the risk of bias in RCTs is lower than other study designs, we initially intended to conduct this study exclusively using RCTs. The number of RCTs that were available was found to be low, and hence, the focus in terms of study design was broadened. Through further explorations, no cohort studies that fit the inclusion criteria were identified, and therefore, published RCTs and CCS on the effects of meditation on telomere length were selected for this review. The articles with titles which contained the exclusion terms: review, short reports and survey were removed by one author prior to the blind title and abstract screening and there were only 11 such articles. Two independent reviewers (ND, NDS) screened and selected the studies, extracted the data and assessed the quality of evidence, and any disagreements were discussed with a third reviewer (NS). Studies reporting on TL in healthy adults, who were experienced/long-term meditators (in CCS) who practised meditation for 3 years and those who received meditation training (in RCTs), regardless of gender and meditation technique, were included. Review criteria related to study population, intervention, comparisons and outcome are detailed in Additional file 2.

The methodological quality of the RCTs was assessed using the Cochrane risk of bias tool [14]. The two RCTs comprised five comparisons were pooled using Review Manager Software (version 5.3). The methodological quality of the CCS was analysed according to the Joanna Briggs Institute critical appraisal checklist for CCS using ten criteria [15].

## Results

**Fig. 1:**
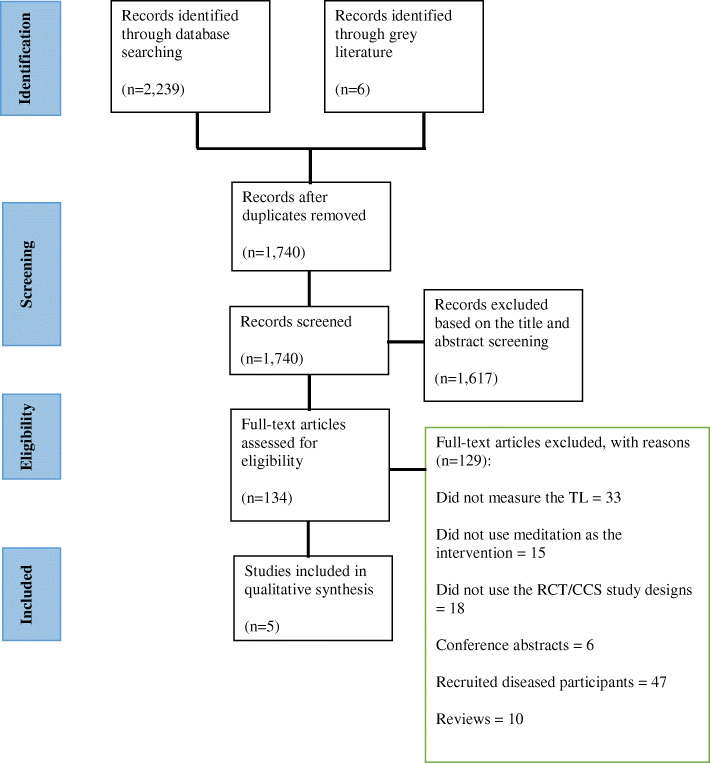
PRISMA flow diagram of study selection for the systematic review

**Fig. 2:**
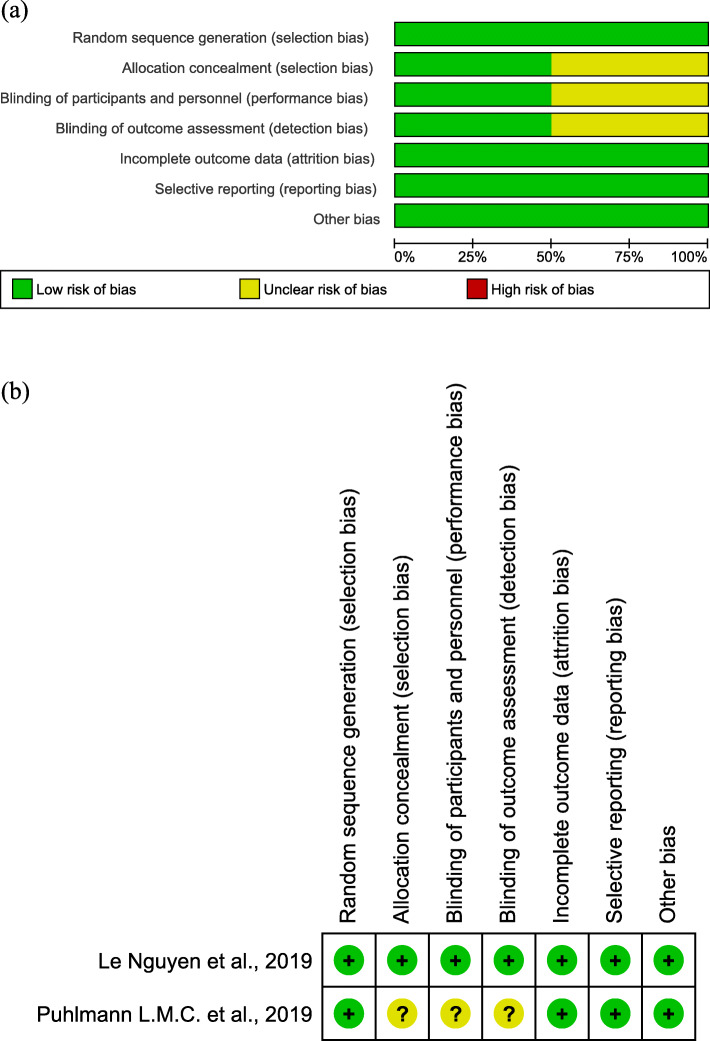
**a** Risk of bias graph and **b** summary of risk of bias of randomized controlled trials

In total, 1740 articles underwent screening and five peer-reviewed, full-text articles, three CCS [16,18] and two RCTs [11, 12] met the full eligibility criteria (Fig. 1). The selected studies were conducted in the USA (*n* = 2), Spain (*n* = 2) and Germany (*n* = 1) and were published between 2013 and 2020. The total number of healthy adult participants examined across all included studies was 615. This comprised 111 in CCS (mean age 48.2 years, 63.46% males) and 504 in RCTs (mean age of 46.7 years, 40.43% males) (see Additional file 3). CCS included participants with long-term meditation experience ranging from 4 [17] to 10 years [16, 18] with a mean of at least 60 min of practice per day [16, 18]. All participants in RCTs received 6 [11] to 12 [12] weeks of meditation training with a mean of 1.5-h small group sessions and daily practice (see Additional file 4). The methodological quality of the studies that were included is shown in Fig. 2 and Additional file 5.

One CCS showed significantly longer TL in meditators compared to controls [16] while another study showed that meditators have longer median TLs relative to controls [17] with the difference being significant in females. The third CCS [18] showed positive beneficial effects of meditation on TL in cases but the difference was not significant (see Additional file 6). One RCT did not show any effects of meditation on leukocyte TL [12]. The other RCT which focused on two different techniques showed a significantly lower decrease in the TL in the subgroup which practised loving-kindness meditation but not mindfulness meditation[11].

**Fig. 3:**
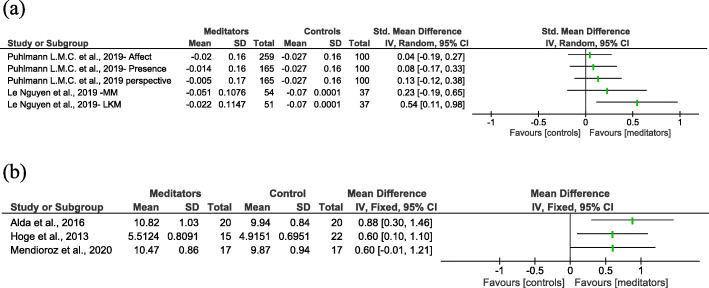
Summary of effect sizes and 95% confidence interval (CI) for telomere length in **a** randomized controlled trials and **b** case-control studies

The assessments related to psychological well-being suggested that expert/long-term meditators showed higher and lower levels of positive and negative attributes, respectively. None of these studies reported any adverse effects of meditation practice. The effect estimates for TL in the RCTs and CCS are shown in Fig. 3a and b, respectively. Certainty of the evidence, assessed based on the GRADE approach, is shown in Additional file 7.

## Discussion

This review provided limited evidence from both observational and interventional studies that meditation and mindfulness practices are likely to reduce shortening of telomeres. One CCS included in this review had shown that in females, meditators had significantly longer telomeres than the comparison group. However, this observed effect may also be due to other factors which differed between the studies such as the duration of meditation practice or other biological factors. Whereas a RCT demonstrated significant changes in TL following only 6 weeks of meditation [11], CCS with those who practised meditation for more than 4 [17] or 10 [18] years failed to show such changes. While long-term practice would be expected to result in more benefits, close matching of participant profiles, commitment and the focused guidance during a short period may have contributed to these outcomes. Although the specific technique of meditation differed among the studies, these techniques are based on a few common underlying approaches such as maintaining attention on a chosen object, thought or activity and training to pay attention to the present moment.

Rigorous criteria ensured that meditation-based practices exclusively were used as the exposure/intervention in the studies included in our review, as use of multiple techniques within a group can confound effects of meditation alone. As the changes in TL were assessed only in healthy adults, the findings of our review are more likely to be applicable to the larger general population. Apart from meditation, lifestyle interventions such as weight loss programmes and practice of yoga have previously shown benefits on TL [19, 20].

Strong religious connotations of meditation in Asian settings may make the practitioners of these techniques wary of collaborating with researchers who adopt a more scientific approach, which can contribute to the dearth of studies reported from these countries. Further, studies conducted in Asian countries and published in other languages would have been excluded by our search criteria. One of the main limitations of this study is lack of a meta-analysis. In a behavioural intervention of this nature where blinding of participants and researchers is generally not feasible, the conventional assessments may unduly downgrade the methodological quality of a study. The effects of meditation is dependent upon how well the participants have learnt and followed a technique, which may be difficult to assess comprehensively and is an inherent limitation of all studies of this nature. Predefined criteria including frequency and duration of meditation, age range and gender, lifestyle and ethnicity are some confounding factors to be taken into consideration in designing future studies.

## Conclusions

The effect of meditation on TL per se is still unclear. Future meticulously designed studies will be required to strengthen the evidence on the beneficial effects of meditation on TL before a strong recommendation can be made for adopting these techniques for modulating the process of cellular ageing.

## Supplementary Information

**Additional file 1: Table 1.** Search strategy for databases.

**Additional file 2: Table 2.** Study selection criteria.

**Additional file 3: Table 3.** Characteristics of the included studies.

**Additional file 4: Table 4.** Details of the interventions included in the review.

**Additional file 5: Table 5.** Methodological quality of Case-Control studies.

**Additional file 6: Table 6.** Summary of findings for the main comparisons.

**Additional file 7: Table 7.** Certainty of the evidences.

**Additional file 8: Table 8.** The list of excluded publications and reasons for exclusion.

## Data Availability

The research data supporting this systematic review and meta-analysis are from previously reported studies and datasets, which have been cited. These prior studies (and datasets) are cited at relevant places within the text as references [11, 12, 16,18].

## Abbreviations

TL: Telomere length
CCS: Case-control studies
RCT: Randomized controlled trials
CI: Confidence intervals
