# Supplementary material for: The effects of meditation on length of telomeres in healthy individuals: a systematic review
Source: Syst Rev. 2021 May 21;10:151. doi: 10.1186/s13643-021-01699-1 (PMC8139075; doi:10.1186/s13643-021-01699-1)
Supplement: Supplementary file 1 — Additional file 1: Table 1. Search strategy for databases. [file 13643_2021_1699_MOESM1_ESM.pdf]

Table 1: Search strategy for databases

Search number	Query
1	“Meditation” AND “Telomere length”
2	“Meditation” AND “Telomere”
3	“Meditation” AND “Telomerase”
4	“Mindfulness” AND “Telomere”
5	“Mindfulness” AND “Telomere length”
6	“Mindfulness” AND “Telomerase”
7	“Healthy Individuals”
8	1 OR 4 AND 7
9	1 OR 6 AND 7
10	2 OR 5 AND 7
11	2 OR 6 AND 7
12	3 OR 4 AND 7
13	3 OR 5 AND 7
14	Limit 13 to RCT AND English
15	Limit 13 to CCS AND English
