# Supplementary material for: The effects of meditation on length of telomeres in healthy individuals: a systematic review
Source: Syst Rev. 2021 May 21;10:151. doi: 10.1186/s13643-021-01699-1 (PMC8139075; doi:10.1186/s13643-021-01699-1)
Supplement: Supplementary file 2 — Additional file 2: Table 2. Study selection criteria. [file 13643_2021_1699_MOESM2_ESM.pdf]

Table 2: Study selection criteria

Study component	Inclusion criteria	Exclusion criteria	Remarks
**Study design**	Randomized controlled trials (RCT) and observational studies	Studies that used other study designs, reviews, and short reports	Since the risk of bias in RCTs is lower than other study modes, it was initially intended to conduct this study exclusively from RCTS. The number of RCTs that were available was found to be low. Hence the focus in terms of study design was broadened to include observational studies. Through further explorations, no cohort studies that fit the inclusion criteria were identified and therefore, published RCTs and case-control studies (CCS) on the effects of meditation on TL were selected for this review.
**Participants**	Healthy adults, who were experienced/long-term meditators (in CCS) and those who received meditation training (in RCTs), regardless of gender and meditation technique were included. (Experienced/long-term meditators were defined as those who had practiced meditation for at least three years.)	Studies conducted on participants with disease conditions such as cancer, Alzheimer’s disease, infertility, obesity, depression, anxiety, stress, and chronic fatigue syndrome were excluded.	
**Intervention**	RCTs, which used any form of meditation technique as the	Studies, which used other stress relieving non-	Although the specific technique of meditation differed among the studies, these techniques are based on a few

	intervention, and CCS that recruited meditators (cases) who have practiced any form of meditation technique were included	meditative techniques such as Yoga and Qigong independently or in combination with meditation techniques were excluded.	common underlying approaches such as maintaining attention on a chosen object, thought or activity and training to pay attention to the present moment. Interventions that differ from the basic techniques and approaches of meditation were excluded to avoid the confounding effects.
**Comparison and outcome**	The primary outcome was TLs between meditators and controls in CCS and the TL difference between pre-post meditation interventions in RCTs. Secondary outcomes were mindfulness and psychological health related variables.	Studies indicating ineligibility through available information were excluded.	
