# Supplementary material for: The effects of meditation on length of telomeres in healthy individuals: a systematic review
Source: Syst Rev. 2021 May 21;10:151. doi: 10.1186/s13643-021-01699-1 (PMC8139075; doi:10.1186/s13643-021-01699-1)
Supplement: Supplementary file 3 — Additional file 3: Table 3. Characteristics of the included studies. [file 13643_2021_1699_MOESM3_ESM.pdf]

Table 3: Characteristics of the included studies

Study	Country of the study	Study design	Study setting	Meditators*	Sample size/sample size calculation	Comparison group*
**Hoge et al., 2013**	USA	Case-control study	Vipassana meditation centers and retreat centers in New England	Experienced meditators over the age of 18, who practiced loving-kindness meditation for more than 4 years regularly and attended at least one overnight meditation retreat.	37/not specified	Control participants were also aged more than 18 and they were not experienced with any meditation and yoga practice. Comparison group matched with mean age (±5 years), gender, education level and levels of depression history.
**Alda et al., 2016**	Spain	Case-control study	Soto Zen Spanish Buddhist community	Experienced meditators aged between 18-65 years, who practiced continuous Zen meditation for more than 10 years with a mean at least of 60 min/per day of formal practice during the entire period.	40/not specified	Controls were relatives, and/or friends of the cases who had a similar lifestyle with the age range between 18-65 years and matched by the age (±2 years), gender and ethnic group.
**Le et al., 2019**	USA	Randomized controlled trial	Durham and Orange county of North Carolina	Healthy midlife adults aged between 35-64 years, who had home internet access, little to no meditation experience, and no current chronic illnesses or disabilities. These participants received the loving-	176/not specified	Waitlist control group, who also had home internet access, little to no meditation experience, and no current chronic illnesses or disabilities. They were also aged between 35-64 years and they did

				kindness/mindfulness meditation training.		not receive any meditation training.
**Puhlmann et al., 2019**	Germany	Randomized controlled trial	The European Community's Seventh Framework program under the ReSource Project	Healthy individuals with the age range of 20-55, who were meditation naïve, and extensively screened for mental and physical illnesses. These participants received the presence, affect, and perspective meditation trainings.	332/not specified	Retest control group with a age range of 20-55 years, who were also meditation naïve, and extensively screened for mental and physical illnesses. They did not underwent all the tests but no meditation training.
**Mendioroz et al., 2020**	Spain	Case-control study	Mindfulness program at the University of Zaragoza and Spanish Association of Mindfulness	Long-term meditators who practiced continuous mindfulness meditation for more than 10 years with a mean at least of 60 min/per day of formal practice during the entire period.	34/not specified	Controls were relatives, and/or friends of the cases who had a similar lifestyle and matched by the age (±2 years), gender and ethnic group.

PCR – Polymerase Chain Reaction

*All Participants were healthy adults
