# Supplementary material for: The effects of meditation on length of telomeres in healthy individuals: a systematic review
Source: Syst Rev. 2021 May 21;10:151. doi: 10.1186/s13643-021-01699-1 (PMC8139075; doi:10.1186/s13643-021-01699-1)
Supplement: Supplementary file 4 — Additional file 4: Table 4. Details of the interventions included in the review. [file 13643_2021_1699_MOESM4_ESM.pdf]

Table 4: Details of the interventions included in the review

Study	Intervention(s)	Duration	Process
Hoge et al., 2013	Loving-kindness meditation	4 years or more	Regular, nearly daily practice<br>Must have attended at least one overnight retreat of 3 days or more
Alda et al., 2016	Zen meditation	More than 10 years	Continuous meditation practice with a mean of at least 60 minutes/day of formal practice
Le et al., 2019	Loving-kindness meditation<br>Mindfulness meditation	6 weeks	Meditation workshop designed to follow health-based format that used six progressive, 1-hour small group sessions with comparable resources and encouragement for individual home practice.<br><br>Participants received homework assignments and five 20-minute audio-recorded guided meditations and were encouraged to cultivate a daily meditation practice, besides the workshop.
Puhlmann. et al., 2019	Presence, affect, and perspective meditation programs<br>(Presence: breathing and body-scan meditation<br>Affect: loving-kindness meditation and affect dyad<br>Perspective: observing thoughts)	3 months for each type	Each meditation program began with a 3-day silent retreat. participants attended weekly 2-hour group sessions and performed the 2 core practices of each session at home for approximately 30 minutes daily for the remainder of the training period
Mendioroz et al., 2020	Mindfulness meditation	More than 10 years	Continuous meditation with a mean of at least 60 minutes/day of formal practice
