# Supplementary material for: The effects of meditation on length of telomeres in healthy individuals: a systematic review
Source: Syst Rev. 2021 May 21;10:151. doi: 10.1186/s13643-021-01699-1 (PMC8139075; doi:10.1186/s13643-021-01699-1)
Supplement: Supplementary file 5 — Additional file 5: Table 5. Methodological quality of Case-Control studies. [file 13643_2021_1699_MOESM5_ESM.pdf]

Table 5: Methodological quality of Case-Control Studies

	Yes Marks = 1	No Marks = 0	Unclear Marks = 0.5	Not applicable
Alda et al., 2016				
Were the groups comparable other than the presence of disease in cases or absence of disease in controls? (disease = meditation)	x			
Were cases and controls matched appropriately?	x			
Were the same criteria used for identification of cases and controls?	x			
Was exposure measured in a standard, valid, and reliable way?	x			
Was exposure measured in the same way for cases and controls?	x			
Were confounding factors identified?	x			
Were strategies to deal with confounding factors stated?	x			
Were outcomes assessed in a standard, valid and reliable way for cases and controls?	x			
Was the exposure period of interest long enough to be meaningful?	x			
Was appropriate statistical analysis used?	x			
Hoge et al., 2013				
Were the groups comparable other than the presence of disease in cases or absence of disease in controls? (disease = meditation)	x			
Were cases and controls matched appropriately?	x			
Were the same criteria used for identification of cases and controls?	x			
Was exposure measured in a standard, valid, and reliable way?	x			
Was exposure measured in the same way for cases and controls?	x			
Were confounding factors identified?	x			
Were strategies to deal with confounding factors stated?	x			
Were outcomes assessed in a standard, valid and reliable way for cases and controls?	x			
Was the exposure period of interest long enough to be meaningful?	x			
Was appropriate statistical analysis used?	x			

Mendioroz et al., 2020			
Were the groups comparable other than the presence of disease in cases or absence of disease in controls? (disease = meditation)	x		
Were cases and controls matched appropriately?		x	
Were the same criteria used for identification of cases and controls?	x		
Was exposure measured in a standard, valid, and reliable way?	x		
Was exposure measured in the same way for cases and controls?	x		
Were confounding factors identified?	x		
Were strategies to deal with confounding factors stated?	x		
Were outcomes assessed in a standard, valid and reliable way for cases and controls?	x		
Was the exposure period of interest long enough to be meaningful?	x		
Was appropriate statistical analysis used?	x		
