# Supplementary material for: The effects of meditation on length of telomeres in healthy individuals: a systematic review
Source: Syst Rev. 2021 May 21;10:151. doi: 10.1186/s13643-021-01699-1 (PMC8139075; doi:10.1186/s13643-021-01699-1)
Supplement: Supplementary file 6 — Additional file 6: Table 6. Summary of findings for the main comparisons. [file 13643_2021_1699_MOESM6_ESM.pdf]

Table 6: Summary of findings for the main comparisons

Study	Sample size/sample size calculation	Cases/ Intervention (n)	Controls (n)	Measure used to assess the TL	Outcomes
**Hoge et al., 2013**	37/not specified	15	22	Real Time PCR	Meditators have median relative longer telomeres (0.39) than controls (0.27)<br>Women meditators had a significantly longer TL (0.42) than controls (0.31) (p = 0.007)
**Alda et al., 2016**	40/not specified	20	20	*In situ* Hybridization	Expert meditators have significantly longer median TL (10.82 kb) compared to the comparison group (9.94 kb) (p = 0.005)
**Le et al., 2019**	176/not specified	LKM - 63<br>MM - 62	51	Real Time PCR	According to the TL difference between pre-post interventions:<br>LKM group - significantly lower decrease in TL (0.048) compared to controls (0.07): (p =0.024)<br>MM group – no TL difference (0.019) compared to control group (0.07)
**Puhlmann et al., 2019**	332/not specified	222	76	Real Time PCR	Control group had 0.004 difference in relative length while the other three interventions, presence, affect and perspective had -0.007, -0.005, and -0.001 TL changes respectively. However, these results were not significant.
**Mendioroz et al., 2020**	34/not specified	17	17	*In situ* Hybridization	Meditators have shown longer TLs (mean = 10.47, SD = 0.86) compared to controls (mean = 9.87, SD = 0.94) without any statistical significance, but showing a strong trend (t = -1.94; df = 32; p = 0.002)

LKM: Loving-Kindness Meditation
MM: Mindfulness Meditation
PCR – Real Time Polymerase Chain Reaction
