# Supplementary material for: The effects of meditation on length of telomeres in healthy individuals: a systematic review
Source: Syst Rev. 2021 May 21;10:151. doi: 10.1186/s13643-021-01699-1 (PMC8139075; doi:10.1186/s13643-021-01699-1)
Supplement: Supplementary file 7 — Additional file 7: Table 7. Certainty of the evidences. [file 13643_2021_1699_MOESM7_ESM.pdf]

Table 7: Certainty of the evidence

Certainty assessment							Certainty
No of studies	**Study design**	**Risk of bias**	**Inconsistency**	**Indirectness**	**Imprecision**	**Other considerations**	
**3**	Observational studies	Not serious[a]	Not serious[b,c]	Not serious[d]	Serious[e]	None	⊕◯◯◯ VERY LOW
**2**	Randomized trials	Not serious[f]	Not serious[b,c]	Not serious[d]	Serious[g]	None	⊕⊕⊕◯ MODERATE

a. CCS are having a high risk of bias compared to RCTs. But when comparing only CCS together, all three CCS fall under low risk of bias.

b. All the studies have shown a positive trend in the effectiveness of meditation on the TL.

c. Heterogeneity of the studies was high since the studies have used different meditation techniques as interventions. But we considered all these techniques as sub techniques of meditation and hence we did not downgrade the grade of evidence.

d. TL was assessed directly using standard methods.

e. Modest sample sizes were used.

f. One study has shown unclear bias to allocation concealment, blinding of participants and personnel (performance bias), and blinding of outcome assessment.

g. Even though the sample sizes were adequate and CI were overlapped, the two ends of the confidence intervals ranged across the no effect line. Therefore, the grade of evidence was downgraded.
