# Supplementary material for: The effects of meditation on length of telomeres in healthy individuals: a systematic review
Source: Syst Rev. 2021 May 21;10:151. doi: 10.1186/s13643-021-01699-1 (PMC8139075; doi:10.1186/s13643-021-01699-1)
Supplement: Supplementary file 8 — Additional file 8: Table 8. The list of excluded publications and reasons for exclusion. [file 13643_2021_1699_MOESM8_ESM.pdf]

Table 8: The list of excluded publications and reasons for exclusions

Title	Reason for exclusion
Carlson, L. E., Zelinski, E. L., Speca, M., Balneaves, L. G., Jones, J. M., Santa, D., … Vohra, S. (2017). Protocol for the MATCH study : Mindfulness and Tai Chi for cancer health A preference-based multi-site randomized comparative e ff ectiveness trial (CET) of Mindfulness-Based Cancer Recovery (MBCR) vs . Tai Chi / Qigong (TCQ) for cancer survivors A . *Contemporary Clinical Trials*, *59*(January), 64–76. https://doi.org/10.1016/j.cct.2017.05.015	Study design: Preference based comparative effectiveness trial<br><br>Intervention: Tai chi – qigong<br><br>Study group: Cancer survivors diagnosed with any type cancer excluding brain, completed primary treatment
Epel, E. S., Puterman, E., Lin, J., Blackburn, E., Lazaro, A., & Mendes, W. B. (2013). *Wandering Minds and Aging Cells*. https://doi.org/10.1177/2167702612460234	Study design: A trait-like aspect of mind wandering based on self-report
Leung, C. W., Laraia, B. A., Bush, N. R., Lin, J., Blackburn, E. H., Adler, N. E., & Epel, E. S. (2016). *Sugary beverage and food consumption , and leukocyte telomere length maintenance in pregnant women*. (February), 1–3. https://doi.org/10.1038/ejcn.2016.93	Study group; pregnant women
Mason, A. E., Hecht, F. M., Daubenmier, J. J., David, A., Lin, J., Moran, P. J., … Epel, E. S. (2019). *Weight loss, weight-loss maintenance, and cellular aging in the Supporting Health through Nutrition and Exercise (SHINE) Study*. *80*(7), 609–619. https://doi.org/10.1097/PSY.0000000000000616.Weight	Intervention: weight-loss program with or without mindfulness training<br>study group: abnormal obesity adults
Nelson, B. W., Allen, N. B., & Laurent, H. (2018). Psychoneuroendocrinology Infant HPA axis as a potential mechanism linking maternal mental health and infant telomere length ☆. *Psychoneuroendocrinology*, *88*(June 2017), 38–46. https://doi.org/10.1016/j.psyneuen.2017.11.008	Study design: Neither RCT or CCS<br>Outcome: mother's and infant’s telomere length
Garland, S. N., & Carlson, L. E. (2015). *The impact of mindfulness-based interventions on symptom burden , positive psychological outcomes , and biomarkers in cancer patients*. 121–131.	Review article

Daubenmier, J., Lin, J., Blackburn, E., Hecht, F. M., Kristeller, J., Maninger, N., … Epel, E. (2012). *Changes in stress , eating , and metabolic factors are related to changes in telomerase activity in a randomized mindfulness intervention pilot study*. https://doi.org/10.1016/j.psyneuen.2011.10.008	Study group: Obese women
Duraimani, S., Schneider, R. H., Randall, O. S., Nidich, S. I., Xu, S., Ketete, M., … Gaylord-king, C. (2015). *Effects of Lifestyle Modification on Telomerase Gene Expression in Hypertensive Patients : A Pilot Trial of Stress Reduction and Health Education Programs in African Americans*. 1–18. https://doi.org/10.1371/journal.pone.0142689	Study group: African American men and women with stage I hypertension
Jacobs, T. L., Epel, E. S., Lin, J., Blackburn, E. H., Wolkowitz, O. M., Bridwell, D. A., … Saron, C. D. (2010). Intensive meditation training , immune cell telomerase activity , and psychological mediators. *Psychoneuroendocrinology*. https://doi.org/10.1016/j.psyneuen.2010.09.010	Outcome: Telomerase activity
Kumar, S. B., Yadav, R., Yadav, R. K., Tolahunase, M., & Dada, R. (2015). *Telomerase Activity and Cellular Aging Might Be Positively Modified by a Yoga-Based Lifestyle Intervention : 00*(0), 1–3. https://doi.org/10.1089/acm.2014.0298	Study design: Case report<br>Outcome: Telomerase activity
A pilot study of yogic meditation for family dementia caregivers Lavretsky, H., Epel, E. S., Siddarth, P., Nazarian, N., Khalsa, D. S., Lin, J., … Irwin, M. R. (2012). *A pilot study of yogic meditation for family dementia caregivers with depressive symptoms : effects on mental health , cognition , and telomerase activity †*. https://doi.org/10.1002/gps.3790	Study population: Family dementia caregivers with depressive symptoms<br><br>Outcome: Telomerase activity
Leung, C. W., Laraia, B. A., Coleman-phox, K., Nicole, R., Lin, J., Blackburn, E. H., … Epel, E. S. (2017). *length maintenance in pregnant women*. *70*(9), 1086–1088. https://doi.org/10.1038/ejcn.2016.93.Sugary	Study population: Pregnant women<br>Study design: Cohort study<br>Intervention: Mindfulness program on psychosocial well-being and gestational weight gain

Ornish, D., Lin, J., Daubenmier, J., Weidner, G., Epel, E., Kemp, C., … Blackburn, E. H. (2008). Increased telomerase activity and comprehensive lifestyle changes : a pilot study. *Lancet Oncology*, *9*(11), 1048–1057. https://doi.org/10.1016/S1470-2045(08)70234-1	Study population: Low-risk prostate cancer<br>Outcome: Telomerase activity
Ho, R. T. H., Ph, D., & Chan, J. S. M. (2012). *A Randomized Controlled Trial of Qigong Exercise on Fatigue Symptoms , Functioning , and Telomerase Activity in Persons with Chronic Fatigue or Chronic Fatigue Syndrome*. 160–170. https://doi.org/10.1007/s12160-012-9381-6	Study population: Chronic Fatigue or Chronic Fatigue Syndrome patients<br><br>Outcome: Telomerase activity
Tolahunase, M., Sagar, R., & Dada, R. (2017). *Impact of Yoga and Meditation on Cellular Aging in Apparently Healthy Individuals : A Prospective , Open-Label Single-Arm Exploratory Study*. *2017.*	Intervention: Yoga<br>Study design: A Prospective, Open-Label Single-Arm Exploratory Study
Tolahunase, Madhuri R, Sagar, R., Faiq, M., & Dada, R. (2018). *Yoga- and meditation-based lifestyle intervention increases neuroplasticity and reduces severity of major depressive disorder: A randomized controlled trial*. https://doi.org/10.3233/RNN-170810	Intervention: Yoga<br>Study population: Major depressive disorder
Carlson, L. E., Beattie, T. L., Giese-Davis, J., Faris, P., Tamagawa, R., Fick, L. J., … Speca, M. (2015). Mindfulness-based cancer recovery and supportive-expressive therapy maintain telomere length relative to controls in distressed breast cancer survivors. *Cancer*, *121*(3), 476–484. https://doi.org/10.1002/cncr.29063	Study population: Breast cancer survivors
Lengacher, C. A., Reich, R. R., Kip, K. E., Barta, M., Ramesar, S., Paterson, C. L., … Park, J. Y. (2014). Influence of Mindfulness-Based Stress Reduction (MBSR) on Telomerase Activity in Women With Breast Cancer (BC). *Biological Research for Nursing*, *16*(4), 438–447. https://doi.org/10.1177/1099800413519495	Study population: Breast cancer patients<br>Outcome: Telomerase activity
Maddux, R. E., Daukantaité, D., & Tellhed, U. (2018). *The effects of yoga on stress and psychological health among employees : an 8- and 16-week intervention study employees : an 8- and 16-week intervention study*. *5806.* https://doi.org/10.1080/10615806.2017.1405261	Study population: Patients, with depression, anxiety and stress and adjustment disorders, and healthy controls

Conklin, Q. A., King, B. G., Zanesco, A. P., Lin, J., Hamidi, A. B., Pokorny, J. J., … Saron, C. D. (2018). Insight meditation and telomere biology: The effects of intensive retreat and the moderating role of personality. *Brain, Behavior, and Immunity*, *70*, 233–245. https://doi.org/10.1016/j.bbi.2018.03.003	Study population: Expert meditators have received a meditation intervention.
Innes, K. E., Selfe, T. K., Brundage, K., Montgomery, C., Wen, S., Kandati, S., … Huysmans, Z. (2018). Effects of Meditation and Music-Listening on Blood Biomarkers of Cellular Aging and Alzheimer's Disease in Adults with Subjective Cognitive Decline: An Exploratory Randomized Clinical Trial. *Journal of Alzheimer's Disease*, *66*(3), 947–970. https://doi.org/10.3233/JAD-180164	Study population: Alzheimer's disease patients
Thimmapuram, J., Pargament, R., Sibliss, K., Grim, R., Risques, R., & Toorens, E. (2017). Effect of heartfulness meditation on burnout, emotional wellness, and telomere length in health care professionals. *Journal of Community Hospital Internal Medicine Perspectives*, *7*(1), 21–27. https://doi.org/10.1080/20009666.2016.1270806	Study design: Cohort study
Tolahunase, M R, Sagar, R., Chaurasia, P., & Dada, R. (2018). Impact of yoga- and meditation-based lifestyle intervention on depression, quality of life, and cellular aging in infertile couples. *Fertility and Sterility*, *110*(4), e67. https://doi.org/10.1016/j.fertnstert.2018.07.203	Study population: Infertility couples<br>Intervention: Yoga
Rao KS, Chakraharti SK, Dongare VS, et al., Antiaging Effects of an Intensive Mind and Body Therapeutic Program through Enhancement of Telomerase Activity and Adult Stem cell Counts. *J Stem Cells*. 2015;10(2):107-125	Outcome: Telomerase activity<br>Study population: Healthy adults and patients
Maddux, R. E., Daukantaité, D., & Tellhed, U. (2018). *The effects of yoga on stress and psychological health among employees : an 8- and 16-week intervention study employees : an 8- and 16-week intervention study*. *5806*. https://doi.org/10.1080/10615806.2017.1405261	Study population: Mild and severe depression patients<br><br>Intervention: Yoga
Keng, S., Yim, O. S., Lai, P. S., Chew, S. H., & Ebstein, R. P. (2019). *Association among dispositional mindfulness , self-compassion , and leukocyte telomere length in Chinese adults*. 1–10.	Outcome: Correlation between Mindfulness and telomere length
